# Revisiting the Use of Quantum Chemical Calculations in LogP_octanol-water_ Prediction

**DOI:** 10.3390/molecules28020801

**Published:** 2023-01-13

**Authors:** Dipankar Roy, Chandan Patel

**Affiliations:** 1 Department of Biological Sciences, University of Alberta, Edmonton, AB T6G 2E9, Canada; 2 Department of Applied Sciences, COEP Technological University, Wellesely Road, Shivajinagar, Pune 411005, Maharashtra, India

**Keywords:** octanol-water partition coefficients, quantum chemical calculations, ab initio methods, density functional theory, continuum solvation models, reference interaction site model

## Abstract

The partition coefficients of drug and drug-like molecules between an aqueous and organic phase are an important property for developing new therapeutics. The predictive power of computational methods is used extensively to predict partition coefficients of molecules. The application of quantum chemical calculations is used to develop methods to develop structure–activity relationship models for such prediction, either based on molecular fragment methods, or via direct calculation of solvation free energy in solvent continuum. The applicability, merits, and shortcomings of these developments are revisited here.

## 1. Introduction

**Figure 1:**
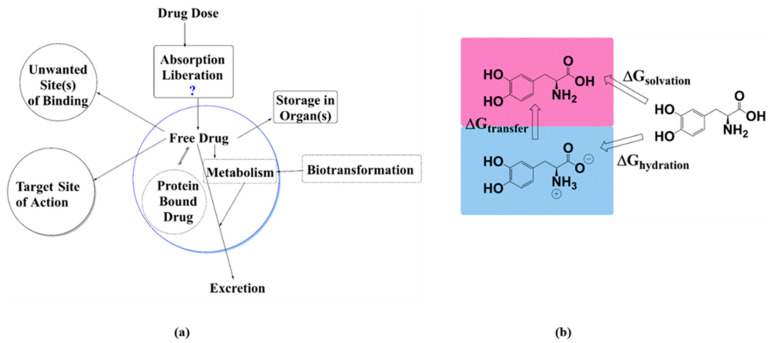
(**a**) Possible fates of a drug molecule inside human body; (**b**) the physical process of distribution of a drug molecule between two phases.

The importance of the accurate prediction of physicochemical properties is quintessential, not only in drug development efforts but also toward applications in material science for molecules with desirable properties. The importance of quantitative structure activity/property relationships (QSAR/QSPR) is undoubtedly essential in chemistry and biology [1,2,3,4]. The linear free energy relationship (LFER) approach often provides a model system based on molecular topologies to relate with biological properties. It is, however, difficult to have a correct QSAR/QSPR for almost all of the cases, although a working relationship is of great demand in the pharmaceutical industry [5,6]. The essential components of such relationships depend on correct knowledge of solvation properties of a target molecule in multiple solvents of interest, with solvent polarities spreading across a broad scale. This is important for determining whether target molecules are adequate to get absorbed in an organ of interest. The important biophysical properties that help a drug to enter the body and distribute across various biological barriers consist of solubility, stability, and permeability [7,8]. The property of interest, lipophilicity, is the ability of a drug or drug-like molecule to dissolve in fat-like substances such as oils or non-polar solvents (toluene, cyclohexane, chloroform etc.). It is well recognized for the successful development of drug candidates, since the introduction by Hansch and Fujita’s method to correlate biological activity with chemical structures [9]. Hydrophobic (water-fearing) drugs prefer to be in the hydrophobic organelles/compartments, such as the lipid bilayers of a cell, while the hydrophilic (water-loving) drugs will prefer to be dissolved in the blood serum (see Figure 1). In general, logP ≤ 0 means high hydrophilicity, and logP > 0 means increased lipophilicity [10]. As per the well-known Lipinski’s “rule of five” for drug-likeness, an orally active molecule has no more than one violation of the following criteria: (i) not more than five hydrogen bond donors (N/O atoms) with one or more hydrogen atoms, (ii) not more than ten hydrogen bond acceptors (N/O atoms), (iii) molecular weight under 500 Daltons, and (iv) logP coefficient of less than five [11]. The final criterion is based on the partitioning of molecules between organic and aqueous phase as in n-octanol/water mixture, traditionally expressed as the negative logarithm of partition coefficient (logP_Organic-Water_ or simply logP) [12]. A study using Caco-2 cell monolayers has shown that the optimal permeability of drugs occurs at ClogP values between 4 and 5, while an increase in ClogP value from this range decreases the permeability of drugs [13].

From a physical chemistry perspective, for non-ionic and neutral forms of compounds, logP is expressed as in Equation (1), where R is the molar gas constant and T is the temperature in K.

$$logP=\frac{-\Delta G_{transfer}}{RT\;ln10},$$

where

Δ*G_transfer_* = Δ*G_solvation_* − Δ*G_hydration_* (1)

Whereas, for ionic compounds, whose overall ionization in solution is pH dependent, often logD is used instead of logP. The logD determines the ratio of the sum of the concentrations of all ionized and unionized forms of the compound in each of the two phases of interest (Equation (2)).

(2) $$logD_{Organic\text{-}Water}=\log\left(\frac{{\left[Solute\right]}_{neutral,Organic}}{{\left[\mathrm{Solute}\right]}_{neutral,Water\;}+{\left[Solute\right]}_{ionic,Water}\;}\right),$$

For molecules whose aqueous ionization constants are known, logD is related to logP via Equation (3). The significance of logD values becomes clear if one looks into the vast range of physiological pH values in the human body, e.g., stomach: ~2; plasma: 7.4; small intestine: ~5–6.8. [14].

(3) $$logD_{Organic\text{-}Water}=logP_{Organic\text{-}Water}-\log\left(1+10^{\left(pH-pK_{a}\right)}\Delta i\right)$$

where Δi=+1 for acids and Δi=−1 for bases [15].

Evidently, the solvation free energy terms are guiding forces here to determine the ability of a molecule to pass through different biological barriers before reaching the target receptor(s). While the distribution of a compound between n-octanol and water mixture (expressed as logP_OW_) was traditionally used as a measure of the lipophilicity and/or hydrophobicity of compounds, strictly speaking the hydrophobicity can be defined as the aggregation propensity of substances in aqueous medium, and n-octanol very poorly represents lipid bilayers. For passive diffusion of drug molecules, which is considered as one of the major pathways of drug absorption, the partition coefficient between aqueous phase and organic phase is directly related to membrane permeability (see Figure 1a for possible fates of a drug inside the human body after ingestion). To gain knowledge of solvation properties, experimental studies are the most reliable; however, for a large molecular dataset, it is a tremendous effort to explore these properties in multiple solvents. This is often not so useful from an industrial point of view, as many compounds fail to clear the initial screening processes based on solvation properties; rendering initial molecular screening expensive. The other and most efficient method is the application of computational methods to explore molecular solvation properties. The use of computational and theoretical tools in understanding molecular solvation is a well-researched area, and is continuously evolving based on need [16]. In the context of logP_OW_ calculation/prediction using theoretical and computational techniques, empirical, semi-empirical, and quantum chemical calculations-based theories have been applied [17,18,19]. The QSAR computed values of logP are often used instead of expensive experimental studies. This is a very effective technique for high throughput virtual screening (HTVS), although they can generate significant inaccuracies, and potential compounds can be rejected, or false positive results may skew efforts [20,21,22].

## 2. Methods for logP Empirical Predictions

The logP prediction methods are broadly classified into two categories, viz. the substructure (or fragment) based methods, and the whole molecule approach. The fragment-based approach is guided by the principle of averaged contributions of simple fragments corrected against large experimental datasets [23,24]. An excellent example of an improved version of this methodology is the clogP program module [25]. The whole molecule approach develops upon molecular lipophilicity potential (MLP), topological indices, and/or molecular quantities to predict logP. While these two methods are complementary to each other as they try to circumvent the shortcomings of each other, they have their own limitations and efficiency considerations [26,27]. For a comparison of *logP* methods on an extensive set of molecules, see references [5,28]. With phenomenal improvements in computer hardware, and with the advent of logic-high programing languages, the scope of implementing and validating QSAR/QSPR models has become a part of routine development [29]. The earlier studies of linear regression analysis were eventually refined using neural networks and genetic algorithms-based computing schemes for logP prediction/validation. The latest in this field is the application of machine learning protocols (random Forest, support vector machine) for building non-linear QSAR/QSPR models and principle component analysis [30,31,32,33].

The solvation properties are a direct outcome of the electronic structure of a molecule, and therefore, electronic structure calculations seem to be an excellent starting point for calculation of logP. The use of quantum chemical methods is applied for two very specific regimes of methods: first, for the development of descriptors to assist in computation of logP using the whole molecule-based approaches; and second, direct calculation of solvation free energy using different continuum solvation models. The following discussion is divided into two sections, viz. applications of quantum mechanics based molecular descriptors for logP prediction, and direct prediction of logP via free energy of solvation calculations.

## 3. Applications of Quantum Mechanics Based Molecular Descriptors for logP Prediction

**Figure 2:**
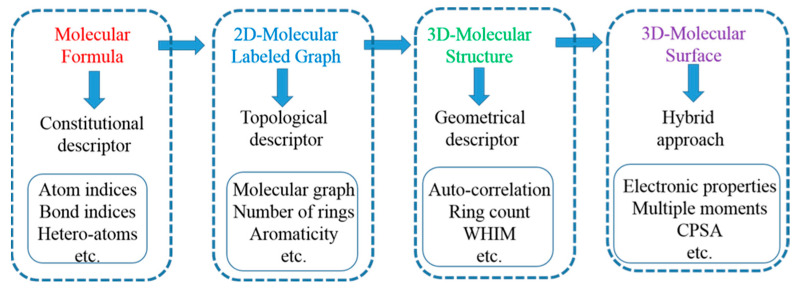
Hierarchical developmental tree of QSAR descriptors for applications in molecular design.

Since the first demonstration by Hanch and Fujita, that logP can be calculated using a linear correlation method, several similar methods were reported for various purposes, mostly for QSAR/QSPR analysis (see Figure 2 for information on different types of descriptors used in logP prediction modules). The Hansch–Fujita method predicts logP of a compound with substitution of H with X, using a relation with the unsubstituted form as *π* = logP*_X_* − logP*_H_* where the parameter π is obtained by regression analysis against standard databases. This simple looking equation leads to a plethora of models involving molecular properties, and more significantly intrinsic electronic properties, which can be partitioned over atoms present in a molecule. The application of empirical and quantum chemical (QC) methods to derive such descriptors have become very popular. The QC methods were more accurate and well suited for calculating atomic partial-charges, molecular orbital (MO) energies, dipole moments, and many other properties. In earlier days, the demand for heavy computational resources to generate molecular wave function had restricted the use of the QM descriptors; however, semi-empirical calculations became quite popular, which were developed within the mathematical framework of molecular orbital theory, but with more generalizations and approximations (SCF-MO). There are several semi-empirical methods available which have been benchmarked against experimental atomic and molecular data to be used for generation of molecular descriptors [34]. The first descriptor to be used for logP prediction were the atomic charge densities. The method is based on building a linear relation for the set of 61 compounds covering hydrocarbons, alcohols, ethers, carbonyls, amines, nitriles, and amides [35]. The squared atomic charges, calculated using semi-empirical MNDO/3 Hamiltonian method, are used to develop a correlation equation as

$$logP=0.344+0.2078n_{H}+0.093n_{C}-2.119n_{N}-1.937n_{O}-1.389\Sigma q_{C}^{2}-\;17.28\Sigma q_{N}^{2}+0.7316\Sigma q_{O}^{2}+2.844NA+0.910NT+1.709NM,$$

resulting in a statistical correlation of n = 61, r^2^ = 0.985, s = 0.15.

The parameters used in this relation are n_H_, n_C_, n_N_, and n_O,_ and represent the numbers of hydrogen, carbon, nitrogen, and oxygen atoms, respectively; q_i_ is the atomic charge on the corresponding atoms; NA, NT, and NM are numbers of carboxy, cyano, and amide groups, respectively. Interestingly, this approach yielded better results than fragment-based methods, despite being simplistic. A subsequent development was made involving curvature of molecular surface, molecular surface area, and dipole moment. This new model predicted logP with very good correlations for 118 molecules [36,37]. For a series of substituted phenols, the logP prediction scheme was reported using HOMO and LUMO energy gaps, molecular volume, total surface area, molar mass, refractivity, melting point, charge on oxygen atom, and polarizability of the molecule, with excellent success [38]. For a larger dataset of 592 molecules, an excellent QSAR model was developed using only polarizability and partial atomic charges on nitrogen and oxygen. This three-parameter based model is simple and covers a vast molecular space [39]. The van der Waal volume was incorporated in a more recent model computed using density functional theory (DFT) based B3LYP, functional with a double-ξ quality basis set. The authors have used multivariate analysis revealing differences between the chemical classes in terms of their electronic properties, and importance of the frontier MOs in logP prediction [40]. A theoretical development of heuristic molecular lipophilicity potential, using quantum chemically calculated electrostatic potential (ESP) as a unified solvent-philicity potential was reported by Du et al. [41]. The resultant linear regression analysis has the form of:

$$logP=\frac{a\times L_{M}+b\times H_{M}}{RT\;ln10}+c$$

where molecular lipophilicity (L_M_) and hydrophilicity (H_M_) indices were calculated using quantum chemical calculation (semi-empirical, ab initio Hartree-Fock, and DFT-B3LYP levels). For an overview of the lipophilicity predictions, based on QSAR models in drug design, please see reference [41]. There are numerous reports on development of QSAR models and logP predictions based on QM-methods, although the basic number and types of molecular descriptors that were employed are limited in number as to make the model easier to compute and accessible. The use of QM-methods has often overcome deficiencies of force-field based computation schemes.

## 4. Direct Prediction of logP via Free Energy of Solvation Calculations

The accuracy of implicit solvation methods for solvation free energy calculations, and the role of molecular conformations are two major limitations that handicap applications of direct logP estimations using electronic structure calculations. The positive sides of implicit solvation models are (i) reduced computation time in comparison to an explicit all atom calculation, (ii) inherent parameterization to reproduce experimental macroscopic properties of small molecules and ions, and (iii) for an arbitrary geometry of solute “…continuum models automatically give configurationally sampled solvent effect” [42]. One should note that in the case of flexible molecules, the single conformation implicit solvation free energy may not correctly represent conformational ensembles in different phases. Amongst implicit quantum chemical solvation models, the most prominent ones are (i) the polarization continuum models based on the extension of the ‘Integral Equation Formalism’ (IEF), (ii) Minnesota solvation models developed by Truhlar, Cramer, and coworkers traditionally designated as SMD, and (iii) COnductor like Screening MOdel for Real Solvents (COSMO-RS). The description of the molecular solvation theory and other derivatives of it are beyond the scope of the present review. In short, all these methods depend on generating a molecular shaped cavity to place the solute molecule inside a solvent continuum. The deviations in calculating molecular electronic properties as well as solvation energy are mostly arising from the choice of atomic radii sets, which varies significantly between different methods, and calculation of electrostatic field. The electrostatic calculation based on the Density Functional Theory (DFT) grid with a cavity radius of ~1.2 × van der Waals (vdw) radii is a reliable starting point for molecules covering diverse molecular space.

The accurate prediction of hydration free energy by Minnesota solvation model (SMn series; n = 5, 6, 8, 12 etc.) was an encouraging step towards using an implicit solvation model for logP prediction, as well as using hydration free energy as a descriptor in drug design [43,44]. In a study by Kolar and coworkers on the accuracy of implicit solvation methods in calculating logP_OW_ of 20 neutral drug molecules, the best performers were SMD and COSMO-RS methods, whereas molecular mechanics (MM) based methods performed poorly [45]. The authors also found significant deviations between computed and experimental logP for flexible molecules, when a single rigid conformation is used for computation. In a recent work, the applicability of the Onsager equation for realistic systems has been questioned [46]. Indeed, the computational complexity in calculating ΔG_solvation_ is often the limiting step for correct prediction of the partition coefficients. The COSMO-RS method can use a priori prediction of solubilities of a large class of compounds. The first report of training COSMO-RS on a 150 drug-like compound database and testing on a pesticides database containing 107 entries provided rms deviation of 0.66–0.61 log units [47]. One of the best applications of COSMO-RS in predicting cyclohexane–water distribution coefficients in SAMPL5 dataset was reported by Klamt and coworkers [48]. Ouimet and Paluch made a reference database from Drugbank for a large number of fragments based on 4-amino quinazoline structure with experimental logP values in order to develop a calibration curve for logP prediction of select SAMPL6 compounds, using the Minnesota solvation models [49]. These authors found building a training set did not improve prediction for the SAMPL6 set. Their best results were from untrained SM8 solvation level calculations at the M06-2X/6-31G(d) level. Kundi and Ho calculated logP of two sets of compounds, viz. 34 organic compounds and 55 fluorinated alkanols and carbohydrates using implicit and explicit solvents [50]. These authors found that implicit solvent models performed better than the explicit solvent models with a mean absolute deviation of 0.6 log units using the COSMO-RS continuum model. The best results in this study are obtained from empirical fragment-based predictions (ALOGP and miLogP) with a mean deviation in the range 0.2–0.4 log units. Fluorinated compounds receive a lot of attention in drug development owing to their use as hydrophobicity modulating moiety (e.g., -CF_3_, *p*-F_3_C-C_6_H_4_-). Very recently, Patel and Roy reported a systematic analysis of the effect of atomic radii on logP calculations of 56 fluorinated drug molecules using DFT functionals [51]. The standard SMD model is found to outperform the conductor like the polarizable continuum model, while used in combination with various density functionals. These authors reported no significant basis set size issues in logP calculations. The use of the dispersion corrected density functionals did not improve logP predictions in comparison to those computed by non-dispersion corrected ones, as reported by Patel and Roy [51].

A direct logP_OW_ calculation for a large set of compounds using semi-empirical quantum chemical methods with dielectric continuum models found significant deviations between computed and experimental data [52]. An improved prediction was obtained by using multiple linear regression analysis based on structural and constitutional features of the molecules in the same database. A back propagating neural network model was proposed by Briendl et al. for logP prediction for 104 organic molecules based on AM1 and PM3 semi-empirical MO calculations [53]. Fizer et al. reported a detailed analysis of the performance of a combination of different electrostatic charge generation schemes with semiempirical, Hartree-Fock, and DFT level computations for hydrophilic/lipophilic index and logP calculations of 50 mono-charged organic ions [54]. For this set of ions, the authors obtained the best results with the semi-empirical AM1 Ford–Wang parametric electrostatic potential charges. The performance of the natural population with DFT also resulted in optimal performances.

Amongst physics-based methods, the three-dimensional reference interaction site model (3D-RISM) theory has also been applied successfully toward prediction of partition coefficients. The theory uses first principle statistical mechanics to describe properties of molecular solvents using modest computational resources. The theory has been extended significantly to apply in quantum-chemical calculation using the RISM-SCF method [55,56]. The RISM-SCF method is used for the calculations of molecular aggregates, solvation free energy, and hydration of organometallic systems [57,58,59,60,61]. Successful applications of the 3D-RISM theory with Kovalenko-Hirata (KH) closure relation in predicting cyclohexane–water partition coefficients for SAMPL5 and logP_octanol-water_ were reported by Kovalenko and co-workers [62,63]. It is obvious at this point that the need for good computational/theoretical solvation models is essential at present to address solvation modeling. The physics-based methods, relying on quantum chemical description of solvation, are far away from direct applications in logP calculation based on pure hydration free energy. Methods such as 3D-RISM(KH) and its variants are a suitable replacement; although, they are often plagued with issues related to calculation convergence, system size, and parallelization.

## 5. Conclusions

It is important to address the suitability of a choice of *logP* calculation/prediction based on a case specific manner. For similar molecular structures to be used for high throughput virtual screening, the empirical *logP* predictions based on atom/fragment addition approaches may often suffice. Thus, QSAR/QSPR approaches are adequate here. However, this is clearly not the case for systems with novel and/or exotic fragments, which is often the case for development of new pharmaceuticals. The descriptor-based methods should be developed with special care so as to have a minimum of five times more compounds in the training database, than the number of molecular descriptors used. The whole molecule-based methods may work very well for such a scenario. It is worth, for such cases, to treat the molecular descriptor in the most accurate way possible. For instance, an error in calculating atomic charges and resultant multiple moments would result in significant deviation of molecular dipole moment from the actual value, and would interfere with all subsequent calculations based on these descriptors. The semi empirical AM1 method-based charges are often a good choice to start with. Further desired improvements should be guided by necessity and available computational resources. The quantum chemical calculations are an excellent choice for specific solvation property calculations and should be used judiciously. For small molecular sizes, electronic structure calculations with explicit solvation methods are the best; moreover, for a relatively larger system size, a hybrid approach is recommended. Deploying a hybrid approach is non-trivial and requires understanding of the amount of explicit solvent molecules to be added per molecule of substrate to represent the solvation shell, before immersing the entire supramolecular structure in a dielectric continuum model. The molecular solvation theory-based methods, such as 3D-RISM, should be properly standardized for their use in different solvents before setting off to use them for large molecular databases [64]. Of course, if experimental results are available for a reasonable number of systems, then multiple methods/models should be evaluated for correctness before finalizing the method of choice.

## Data Availability

Not applicable.
